# From Snoring to Soaring: Unveiling the Positive Effects of Continuous Positive Airway Pressure Ventilation on Cardiovascular Health in Patients With Obstructive Sleep Apnoea Through a Systematic Review

**DOI:** 10.7759/cureus.45076

**Published:** 2023-09-12

**Authors:** Shivling S Swami, Soe Lwin Aye, Yash Trivedi, Zoryana Bolgarina, Heet N Desai, Mithum Senaratne, Lubna Mohammed

**Affiliations:** 1 Internal Medicine, California Institute of Behavioral Neurosciences & Psychology, Fairfield, USA; 2 Obstetrics and Gynecology, California Institute of Behavioral Neurosciences & Psychology, Fairfield, USA

**Keywords:** osa pathophysiology, cpap adherence, osa treatment, obstructive sleep apnea (osa), continuous positive airway pressure (cpap)

## Abstract

Increased cardiovascular (CV) morbidity and death are linked to obstructive sleep apnoea (OSA). The primary method of treating OSA is continuous positive airway pressure (CPAP). CPAP has some debatable outcomes on CV events in people suffering from OSA. The current study investigates how CPAP affects CV outcomes. The goal is to evaluate CPAP's effectiveness in lowering CV outcomes in OSA patients. We used a computer to search the PubMed, PubMed Central Library, Science Direct, and Google Scholar databases for studies comparing the effects of CPAP and a control group on CV outcomes in OSA patients. These included randomised control trials (RCT), narrative reviews, systematic reviews, case-control studies, observational studies and meta-analyses. A total of 52,937 patients were included in the final analysis of six RCTs, four observational studies, 10 meta-analyses, one case-control study, two systematic reviews and one narrative review. The weighted mean follow-up lasted for a period of between three months and nine years. The risk of major cardiovascular adverse events (MACE) was the same for both the CPAP and control groups. According to subgroup analysis, patients with lower MACE adherence rates (four hours per night) were more likely to use CPAP. The risk of all-cause mortality, CV-related complications causing mortality, acute myocardial infarction acute stroke, or hospitalisations for angina was the same in the CPAP and control groups. The primary outcome was that in patients with therapy with CPAP in addition to usual care and usual care alone did not prevent CV events in patients with moderate-to-severe OSA and existing CV illness. Patients with OSA who utilise CPAP may not experience fewer CV events. Patients who use CPAP consistently (four hours per night) could benefit from improved CV results. Future research must assess how well-adherent patients with severe OSA and low CV event rates respond to CPAP therapy. In patients who use CPAP for more than four hours each night, CPAP therapy may minimise the risk of MACE and stroke. Additional randomised trials requiring adequate CPAP time adherence are needed to support this perception. Despite the fact that there is no evidence to support the claim that CPAP therapy improves CV outcomes, bias difficulties, CPAP adherence problems, and the patient groups included in each RCT may have made it more difficult to generalise the findings to all patients. Future research is therefore needed to look at these relevant results.

## Introduction and background

Obstructive sleep apnea (OSA) is the most prevalent disorder related to sleep, which is linked to the recurrent collapse of the upper airway during sleeping [1]. OSA affects between 3% and 4% of the general population [2]. According to estimates, the syndrome affects 15% of women and 10% of males [3]. It also affects between 40% and 60% of people with cardiovascular disease [1]. It has been identified as one of the most common risk factors for resistant hypertension [4]. It is common in patients with cardiovascular issues and it is more common in people with coronary artery disease (CAD) than in the general population, with a reported incidence of 38% to 65% [5]. While you sleep, multiple instances of shallow breathing or apnoea are signs of sleep apnoea. In addition to being linked to high blood pressure, oxidative stress, inflammation, and hypercoagulation, sleep apnoea may result in episodic hypoxemia [5]. According to recent research, cardiovascular disease risk may increase if OSA is prevalent. The combined effect of cardiovascular risk factors in the two diseases, such as age, being overweight, smoking, and being sedentary, can explain, at least in part, the connection between OSA and cardiovascular disease [3]. Observational studies have shown a link between sleep apnoea and the risk of major cardiovascular events like CAD, heart failure, stroke, and sudden death has increased [5]. Due to the significant risk of recurrent cardiovascular events among these patients despite modern medications, continuous positive airway pressure (CPAP) may be an effective supplementary treatment for stopping these occurrences [2]. By serving as a pneumatic splint on the upper airway whilst you sleep and relieving the obstruction, CPAP increases the quality of life and decreases the daytime fatigue of OSA patients [2]. According to research by Kiely and colleagues, >20% of hypertensive individuals have OSA, whereas >50% of those with OSA also have hypertension. According to one study, OSA is a significant, recognisable cause of hypertension. CPAP is a well-known OSA treatment that reduces hypoxemic episodes and eases symptoms [1]. Observational studies in diverse subgroups of CAD patients have demonstrated an elevated risk of recurrent cardiovascular events and OSA. For symptomatic OSA patients, CPAP is advised [5]. According to a prior study, OSA dramatically raises cardiovascular morbidity and death, particularly in people with cardiovascular disease. According to several studies, CPAP lowers systolic and diastolic blood pressure in patients with OSA by decreasing OSA-related central sympatho-excitatory activity [4]. CPAP therapy has been shown in randomised controlled trials (RCTs) to improve endothelial function, raise insulin sensitivity, and systolic blood pressure by six to seven millimetres of mercury (mmHg) in individuals with persistent hypertension and by two to three mmHg in individuals who have normotensive OSA [5]. Studies from population-based cohorts and sleep clinics have linked OSA to cardiovascular events, including stroke. According to observational studies, CPAP therapy helps individuals with severe and their risk of fatal and nonfatal cardiovascular events is reduced by OSA [2]. Using CPAP is associated with a decreased incidence of cardiovascular issues and cardiovascular-related fatalities, according to clinical observational research., especially in treatment-adherent patients [5]. Although using CPAP therapy for OSA patients is related to better blood pressure and glycaemic management, CPAP's impact on cardiovascular events in OSA patients remains controversial. Even while some randomised studies indicated no advantage for CPAP in lowering cardiovascular outcomes, previous meta-analyses suggested that poor CPAP adherence may be to blame for the lack of good consequences [1]. However, due to the high prevalence of cardiovascular issues in people with OSA, other specialists advise the long-term usage of CPAP treatment for all patients with sleep-disordered breathing, regardless of daytime symptoms [2]. There are still questions regarding the relationships between CPAP and vascular disease and death, even though numerous trials have shown the connections between positive airway pressure (PAP) with cardiovascular outcomes. Individual trial results have also been erratic. Although there have been previous meta-analyses, they have yet to consider the significant new data and insights provided by the big Sleep Apnoea Cardiovascular Endpoints (SAVE) experiment [6]. The SAVE experiment investigated the effects of CPAP therapy in patients with clinically significant cardiovascular (CV) diseases such as coronary artery disease, stroke and moderate-to-severe OSA to address this issue. The SAVE study found no evidence that CPAP therapy decreased the incidence of cardiovascular events, with a mean CPAP usage time of 3.3 hours per night (hazard ratio (HR) 1.10, 95% confidence interval (CI) 0.91-1.32; P = 0.34). A non-significant trend favouring the CPAP arm was observed in the same experiment when data were corrected for more excellent compliance time (four hours per night) (HR 0.80, 95%CI 0.60-1.07; P = 0.13). With enough nightly CPAP duration (four hours per night), Barbe and colleagues and the randomised intervention with CPAP in CAD and sleep apnoea trial also discovered a similar favourable trend [7,8]. Despite existing medications, there have been reports of recurrent cardiovascular events among patients with OSA; CPAP may be a different helpful treatment to stop these occurrences. In clinical trials evaluating its efficacy in primary and secondary cardiovascular prevention, CPAP therapy was not found to significantly reduce the occurrence or recurrence of cardiovascular events. This article examines the relationship between OSA and cardiovascular risk in addition to reviewing recent clinical studies, systematic reviews, and meta-analyses on the effectiveness of CPAP in primary and secondary cardiovascular prevention. Type II diabetes mellitus and OSA usually coexist together as per estimates from clinics, type II diabetics with OSA have a prevalence of 18% to 36% [1-3], and 50% of OSA patients also have type II diabetes or impaired glucose metabolism [9]. Early CPAP therapy improves long-term survival in individuals with ischemic stroke with moderate-severe OSA [10]. We conducted this systematic review to examine all of the information that was available and determine whether CPAP reduces cardiovascular outcomes in people with OSA.

## Review

Methods 

This systematic review seeks to study and assess the use of CPAP in patients with OSA to prevent negative cardiovascular consequences. This systematic review was conducted in accordance with the Preferred Reporting Items for Systematic Reviews and Meta-Analyses (PRISMA) 2020 guidelines.

Search Strategy

The past 10 years' worth of pertinent articles were chosen after a thorough search of databases including PubMed, PubMed Central (PMC), Science Direct Library, and Google Scholar for articles on Cohort Studies, Systematic Reviews and Meta-Analysis, Observational Studies, Narrative Reviews, and Randomised Control Trials (RCTs). The terms "CPAP," "Obstructive Sleep Apnoea," and "Cardiovascular Events" were used to construct the search, which was then combined using the Boolean operators "AND" and "OR." The published articles were reduced using a mesh technique. The databases checked for article collections are shown in Table 1 along with the search method employed.

**Table 1:** Databases used for collecting articles (along with search strategies and appropriate filters). CPAP: Continuous Positive Airway Pressure; PMC: PubMed Central

Type of database	Keywords	Search strategy	Filters used	No. of records
PubMed	Cardiovascular disorders CPAP Sleep apnea Obstructive Systematic Review	"Continuous Positive Airway Pressure"[Majr]AND( "Cardiovascular Diseases/complications"[Majr] OR "Cardiovascular Diseases/drug therapy"[Majr] OR "Cardiovascular Diseases/mortality"[Majr] OR "Cardiovascular Diseases/pathology"[Majr] OR "Cardiovascular Diseases/physiopathology"[Majr] OR "Cardiovascular Diseases/prevention and control"[Majr] OR "Cardiovascular Diseases/therapy"[Majr] )AND( "Sleep Apnea, Obstructive/complications"[Majr] OR "Sleep Apnea, Obstructive/diet therapy"[Majr] OR "Sleep Apnea, Obstructive/drug therapy"[Majr] OR "Sleep Apnea, Obstructive/mortality"[Majr] OR "Sleep Apnea, Obstructive/rehabilitation"[Majr] OR "Sleep Apnea, Obstructive/therapy"[Majr] )	Full-text Meta-Analysis Observational Study Randomized Controlled Trial Systematic Review 10 years Humans	89
PMC	Cardiovascular disorders CPAP Sleep apnea Obstructive Systematic Review	((continuous positive airway pressure (CPAP)) AND (cardiovascular events)) AND (obstructive sleep apnea)	Full-text Meta-Analysis Observational Study Randomized Controlled Trial Systematic Review 10 years Humans	82
Science Direct	Cardiovascular disorders CPAP Sleep apnea Obstructive Systematic Review	CPAP AND Cardiovascular outcomes AND Obstructive Sleep Apnea	Published in the last 10 years Review Articles Research Articles	14
Google Scholar	Cardiovascular disorders CPAP Sleep apnea Obstructive Systematic Review	CPAP AND Cardiovascular outcomes AND Obstructive Sleep Apnea	Published in the last 10 years	34

Inclusion criteria and exclusion criteria: The studies were picked for inclusion based on the following participant, intervention, and outcome characteristics: Participants: population with OSA symptoms that spans all ages, genders, and ethnicities. The use of CPAP in the aforementioned demographic is the intervention. Papers written and published in English and translated papers, papers focusing on all age groups, papers focusing on CPAP including outcomes focused on cardiovascular events in patients with OSA, RCTs, cohort studies, observational studies, systematic reviews, narrative reviews, meta-analyses and case-control studies. Animal studies patients, translated papers, grey literature, irrelevant studies, and studies that do not address the research question or topic of interest. Studies that focus on a different Population, Intervention, Comparison, or Outcome (PICO) than specified in the review. Case reports or case series, conference abstracts or posters without full-text articles. Studies that do not investigate the specified intervention or comparator(s) of interest. Studies that do not report outcomes relevant to the research question or fail to measure key outcomes. Studies with incomplete or insufficient data extract the required outcome measures. Studies with a high risk of bias or poor methodological quality, as assessed by appropriate quality assessment tools. Studies with incomplete or inadequate reporting of methods, results, or statistical analyses.

Results

**Figure 1:**
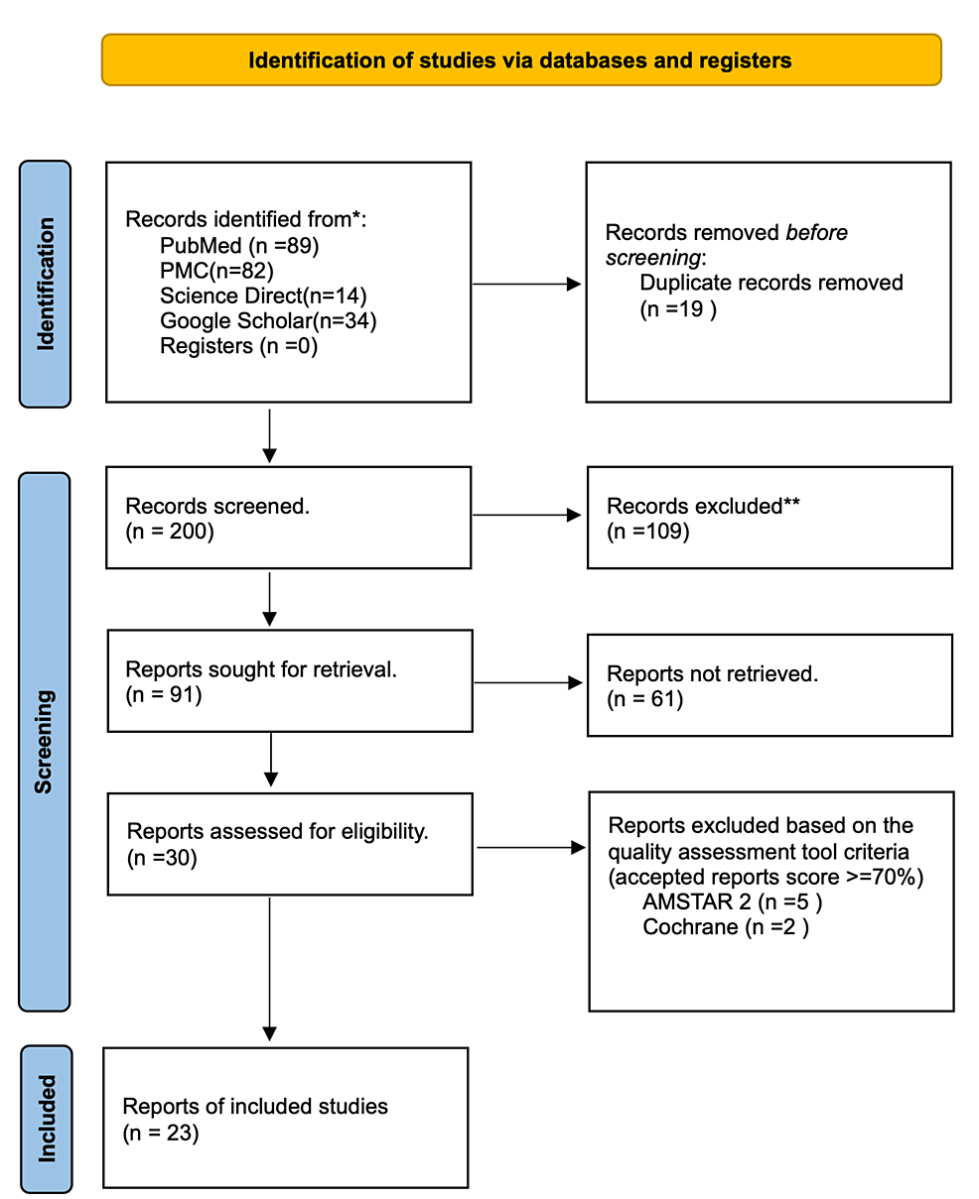
PRISMA flow chart illustrating the screening process and quality assessment of the articles. PRISMA: Preferred Reporting Items for Systematic Reviews and Meta-Analyses; PMC: PubMed Central; AMSTAR 2: Assessment of Multiple Systematic Reviews 2

Using the aforementioned search techniques and proper filters, 214 articles from the databases that have been published within the last 10 years (January 2013 to May 2023) were found, utilising Google Scholar's 2013-2023 filter and PubMed's Meta-Analysis, Observational Study, RCTs, Systematic Reviews and Meta-analysis, Human research, English and translated publications filters, respectively. The titles and abstracts of the papers were then used to screen them. The eligibility requirements and availability of the full text were used to filter the articles. Following the removal of duplicates and irrelevant records, the screened articles were subjected to quality assessment using tools such as the Newcastle-Ottawa checklist for case-control and cohort studies, the Cochrane (CCBRT) for RCTs and non-RCTs, and Assessment of Multiple Systematic Reviews 2 (AMSTAR 2) for systematic reviews and meta-analyses. The PRISMA chart, as seen in Figure 1.

The studies included in this systematic review are summarised in Table 2.

**Table 2:** Included studies CPAP: Continuous Positive Airway Pressure; OSA: Obstructive Sleep Apnea; MACE: Major Cardiovascular Adverse Effect; CAD: Coronary Artery Disease; SBP: Systolic Blood Pressure; DBP: Diastolic Blood Pressure; MI: Myocardial Infarction; CVE: Cardiovascular Events; PAP: Positive Airway Pressure; AMSTAR: Assessment of Multiple Systematic Reviews; RCT: Randomised Control Trials; CAD: Coronary Artery Disease; CV: Cardio Vascular; ESS Epworth sleepiness scale; BP: Blood Pressure; ITT: Intention to treat; RH: Resistant Hypertension; ACS: Acute Coronary Syndrome; mmHg: millimetres of mercury

Author	No. of participants	Study type	Study subjects with	Conclusion	Follow-up period	Quality assessment tool and score
Ayman Elbadawi et al. [1]	N = 5684	Meta-Analysis	CPAP&OSA	No significant difference between the CPAP and control groups in occurrence of MACE at a mean follow up of 42.6 months; On subgroup analysis, CPAP use was associated with lower MACE among the CPAP-adherent subgroup (≥four hour/night), compared with CPAP non-adherent subgroup.	Mean follow-up was 42.6 months	AMSTAR-12/16
Ferran Barbé et al. [2]	N = 725	Randomized Control Trial	CPAP and Hypertension and Cardiovascular events	In patients with OSA without daytime sleepiness, the prescription of CPAP compared with usual care did not result in a statistically significant reduction in the incidence of hypertension or cardiovascular events. However, the study may have had limited power to detect a significant difference.	Mean duration of three years	Cochrane- 5/7
Resano Barrio et al. [3]	N =11,233	Systematic Review	OSA; CPAP; Cardiovascular Risk	Epidemiological and clinical evidence indicates that OSA may be a potentially modifiable risk factor for arterial vascular disease. OSA has been associated with a higher incidence of hypertension and cardiovascular disease. However, clinical trials on the efficacy of CPAP in primary and secondary cardiovascular prevention have not demonstrated a significant reduction in the incidence and/or recurrence of cardiovascular events.	Mean weighted follow-up period was of six years	AMSTAR-13/16
Zhiwei Huang et al. [4]	N = 83	Randomized Control Trial	CPAP or Blood Pressure or Hypertension or Coronary Heart disease	Significant Reduction of SBP In CPAP group, SBP decreased by eight mm Hg, Hypertension control was improved however DBP did not reach any statistical significance.	Mean duration of follow-up was 36 months	Cochrane-7/7
Xiao Wang et al. [5]	N =1430	Systematic Review and Meta-Analysis	CPAP; CAD; OSA	Treatment with CPAP was associated with a significantly lower risk of MACE in six observational studies but this was not reproduced in two RCTs CPAP significantly reduced the risk of all-cause death (four observational studies) and cardiovascular death (three observational studies), which were also not confirmed in RCTs.	Mean follow-up duration was from 36 to 86.5 months.	AMSTAR-14/16
R. Doug McEvoy et al. [6]	N = 2717	Randomized Control Trial	CPAP; Cardiovascular Events; OSA	No significant effect on any individual or other composite cardiovascular end point was observed. CPAP significantly reduced snoring and daytime sleepiness and improved health-related quality of life and mood.	Mean follow-up of 3.7 years	Cochrane- 5/7
Jie Yu et al. [7]	N = 7266	Systematic review and meta-analysis	CPAP; OSA; Cardiovascular Events	No significant association of PAP compared with no treatment on a composite outcome of acute coronary syndrome events, stroke, or vascular death. There was also no significant association with individual outcomes or all-cause death.	_	AMSTAR- 12/16
Safi U Khan et al. [8]	N = 4268	Systematic review and meta-analysis	CPAP; Cardiovascular events; OSA	CPAP therapy might reduce MACE and stroke among subjects with CPAP time exceeding four hour/night. Additional randomized trials mandating adequate CPAP time adherence are required to confirm this impression.	Mean follow-up of 37 months	AMSTAR- 13/16
Yasha Chen et al. [9]	N = 2590	Systematic review and meta-analysis	CPAP; Cardiovascular events; OSA	The results were nearly the same in individual outcomes including all‐cause death and cardiovascular death. CPAP is associated with at least 37% decrease in the risk of MACE compared to control patients in usual care group. Yet our analysis demonstrated null result with respect to MI, stroke or repeat revascularization.	Mean follow‐up was 57.6 months from 36 months to 86.5 months	AMSTAR- 14/16
Gonzalo Labarca et al. [10]	N = 5817	Systematic Review and Meta-Analysis	CPAP; Sleep apnea; Obstructive Meta-analysis	Although there is no evidence that CPAP therapy improves CV outcomes, concerns regarding risk of bias, CPAP adherence, and the population included in each RCT may have reduced the strength of the findings to support the benefit in all patients, and future research exploring these relevant outcomes is needed.	Mean follow up ranged from six to 84 months.	AMSTAR- 14/16
Felipe da Silva Paulitsch et al. [11]	N = 3314	Systematic Review and Meta-Analysis	Obstructive sleep apnea; Continuous positive airway pressure; Cardiovascular disease; Meta-analysis	Low-quality evidence suggests that CPAP therapy does not significantly improve survival or prevent major cardiovascular events in adults with OSA and cardiovascular disease.	Mean Follow-up varied from three to six years	AMSTAR- 14/16
Ahmed S. Abuzaid et al. [12]	N= 3,780	Meta-Analysis	Obstructive sleep apnea; Continuous positive airway pressure; Cardiovascular disease Meta-analysis	In conclusion, compared with medical therapy alone, utilization of CPAP in patients with OSA is not associated with improved cardiac outcomes except in patients who wore it for more than four hours.	Mean follow-up of 3.7 years	AMSTAR- 12/16
Jun Guo et al. [13]	N = 4146	Meta-Analysis	Obstructive sleep apnea; Continuous positive airway pressure; Cardiovascular disease Meta-analysis	CPAP therapy was associated with a trend of decreased risk of cardiovascular events. Furthermore, ESS and BP were significantly lower in the CPAP group. Larger randomized studies are needed to confirm these findings.	Mean follow-up of 30.5 months	AMSTAR- 12/16
Yingke Zhao et al. [14]	N = 3,008 participants	Meta-Analysis	Obstructive sleep apnea; Continuous positive airway pressure; Cardiovascular disease Meta-analysis	In conclusion, OSA can independently increase the risk of cardiovascular events, even after adjustment for confounders. Sleep health should be given utmost importance due to its extensive influence on cardiovascular disorders.	Mean follow up was of three months	AMSTAR- 12/16
Christine Parsons et al. [15]	N = 5,000	Systematic review	Obstructive sleep apnea; Continuous positive airway pressure Cardiovascular disease	The majority of studies found no significant improvement in cardiovascular outcomes with CPAP, although many noted nonsignificant benefits. Adjusted analysis in several trials showed significant cardiovascular benefit in those patients with higher CPAP compliance. Existing trials may lack sufficient follow-up and CPAP compliance, among other limitations.	Mean follow-up of nine years	AMSTAR- 12/16
Miguel-Angel Martínez-García et al. [16]	N = 194	Randomized Control Trial	CPAP; Blood Pressure; OSA; Resistant Hypertentsion	The primary end point was the change in 24-hour mean blood pressure after 12 weeks. Secondary end points included changes in other blood pressure values and changes in nocturnal blood pressure patterns. Both ITT and per-protocol analyses were performed.	Mean follow-up of three months	Cochrane- 5/7
Yuksel Peker et al. [17]	N=622	Randomized Controlled Trial	Obstructive sleep apnea; Coronary artery disease; Cardiovascular outcomes	Routine prescription of CPAP to patients with CAD with non-sleepy OSA did not significantly reduce long-term adverse cardiovascular outcomes in the intention-to-treat population. There was a significant reduction after adjustment for baseline comorbidities and compliance with the treatment.	Mean follow-up was 57 months	Cochrane- 5/7
Yüksel Peker et al. [18]	N=260	Observational	Obstructive Sleep apnea; Coronary artery disease; Cardiovascular Outcomes	We conclude that the risk for MACCEs was not increased in CAD patients with sleepy OSA on CPAP compared with patients without OSA.	Mean follow-up was 57 months.	New Castle Ottawa- 7/9
Yüksel Peker et al. [19]	N=343	Randomized Controlled Trial	Obstructive Sleep Apnea; Coronary Artery Disease; Cardiovascular Outcomes	We conclude that OSA is an independent risk factor for adverse cardiovascular outcomes in patients with ACS. CPAP treatment may reduce this risk, if the device is used at least four h/day.	Mean 4.7-year follow-up	Cochrane- 5/7
Roxana Pleava et al. [20]	N= 33	Observational Study	Sleep apnea; Resistant Hypertension; Body-Mass Index; CPAP therapy	In our cohort of OSA patients with RH, long-term adherence to CPAP therapy was associated with weight loss and improvement in cardiac rhythm outcomes.	Mean follow-up period of four years.	New Castle Ottawa- 7/9
Ryutaro Shirahama et al. [21]	N= 918	Observational Study	Sleep disorders; Outcomes research; Cardiac device therapy	Long-term, good CPAP therapy adherence was associated with lower diastolic blood pressure without significant weight loss.	Mean follow up period of 24-months.	New Castle Ottawa- 7/9
Cristina Navarro-Soriano et al. [22]	N= 163	Observational Study	Stroke; Sleeep Apnea; CPAP; Cardiovascular Events ;Coronary artery disease	In patients with RH and moderate-severe OSA, an ineffective treatment with CPAP showed a trend toward an increase in the incidence of CVE (particularly neurovascular events and hypertensive crises) without any changes with respect to coronary events.	Mean follow up period was 58 months	New Castle Ottawa- 7/9
Francisco Garcia-Rio et al. [23]	N=288	Case Control Study	Sleep apnea; Myocardial infarction; Prognosis; Revascularization; CPAP	Mild-severe OSA is an independent risk factor for MI. Risk of recurrent MI and revascularization was lower in OSA patients who tolerated CPAP.	Mean follow up period was five-eight years	New Castle Otttawa 7/9

Discussion

Pathophysiology of OSA

**Figure 2:**
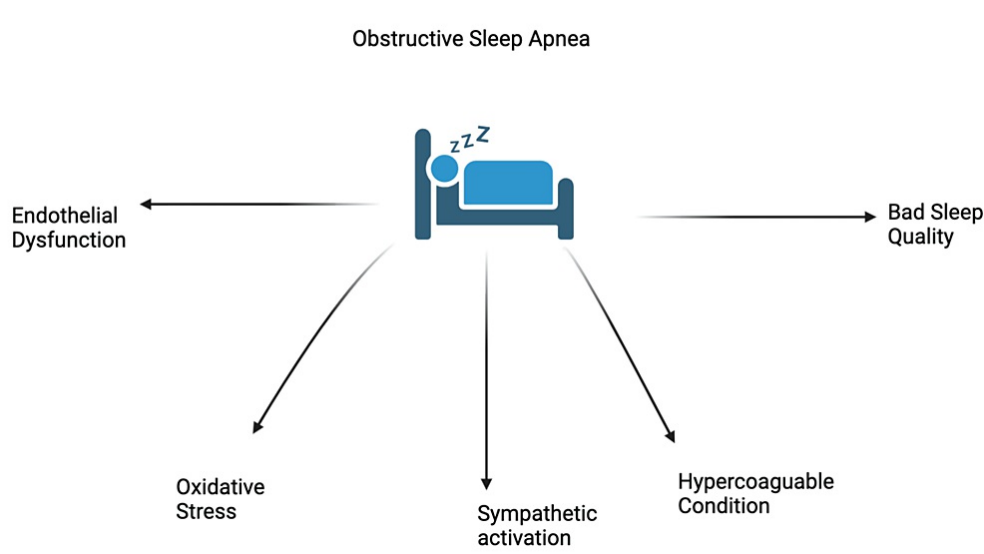
Illustrating various pathophysiologic disturbances caused by obstructive sleep apnea. Created by Shivling Swami

The underlying causes of OSA and its adverse effects on cardiovascular outcomes are still unknown [9]. Several pathophysiological pathways have been linked to the increased cardiovascular events associated with OSA. The primary pathogenic factors that trigger intermittent airway blockage are likely intra-thoracic pressure swings and the resulting hypoxemia. As a result, endothelial dysfunction, oxidative stress, sympathetic activation, and a hypercoagulable condition develop owing to an elevated systemic inflammatory response. This relationship between the pathophysiology of apnoea and hypopnea and the development of coronary artery disease (CAD) seems intriguing [1]. Disturbances in brain auto-regulation and a pro-atherogenic state are some other hypothesised explanations [1]. These pathophysiological modifications may raise nighttime blood pressure and heart rate, myocardial oxygen demand and cardiac stress, which may eventually result in cardiovascular events [9]. The following points have been illustrated in Figure 2.

Obesity and OSA are interdependent, which leads to a vicious cycle in which the former contributes to weight increase. At the same time, the latter furthers the former's role in the pathophysiology of OSA. In general, two main pathways contribute to weight gain: OSA's detrimental effects on energy expenditure and the dysregulation of the hormones that regulate hunger and fullness. Hormonal alterations promote weight gain by ghrelin-stimulating hunger and leptin resistance-induced low satiety [9]. Among the people who have OSA, 5% of them have atrial fibrillation (AF) which is also the most prevalent arrhythmia in the general population. Many reasons account for the frequent co-occurrence of OSA and AF. However, the most significant is the nighttime sympathetic tone surges and the rise in systemic hypertension. The higher risk of AF in OSA patients may also be attributed to structural alterations in the left atrium that are independently linked to arterial stiffness. Comparing the CPAP group to the non-CPAP group, scientifically, the CPAP group's baseline arrhythmia prevalence was higher [20]. Cardiovascular disease is caused by OSA by a complex, multifaceted mechanism. Repeated apnoeic episodes cause sympathetic activity to rise, affecting peripheral blood vessels and causing vasoconstriction. Even during normal daytime wakefulness, this increased sympathetic drive is still present. Patients with OSA have reduced heart rate variability and higher blood pressure variability compared to typical participants, both of which have been linked to the development of hypertension and subsequent end-organ problems [15,24-30]. Hypoxemia and sleep deprivation are likely connected to OSA's ability to cause systemic inflammation. There is still controversy over whether the increase in C-reactive protein in OSA patients indicates an underlying inflammatory process or is just attributable to the elevated body mass index (BMI) that frequently co-occurs in this population [15,31]. Despite obesity, some investigations have found a connection between OSA and C-reactive protein levels [15]. Additionally, hypoxemic stress causes the release of regional vasoactive molecules, such as endothelin, a potent vasoconstrictor. In extensive clinical trials, notably in hypertensive patients, a substantial correlation between sleep apnoea and decreased arterial flow-mediated dilation (a surrogate measure of endothelial dysfunction) has been identified. Again, whether this connection exists independently of fat is still being determined. Endothelial dysfunction may emerge due to oxidative stress, vasoconstriction, inflammation, and sympathetic activity. Vasoactive hormone overproduction, elevation of inflammatory mediators, and hypercoagulability are all signs of endothelial dysfunction and risk factors for cardiovascular diseases such as hypertension, heart failure, and cardiac arrhythmias. The relationships between OSA, hypertension, and other cardiovascular events such as stroke shown in observational studies [15,28] may thus be explained by the correlations above. In the myocardium after ischemia, hypoxia-inducible factor-1 (HIF-1) is increased and causes adaptive alterations such as ischemic preconditioning and angiogenesis. This suggests that the persistent intermittent hypoxia that underlies OSA may benefit the myocardium. Reports suggest that persistent hypoxia, a tissue develops tolerance to future hypoxic stimuli above the damage threshold when an insult below the damage threshold is delivered. Additionally, patients with OSA had increased formation of coronary collateral vessels following total coronary blockage. However, these short-term "beneficial" benefits of intermittent hypoxia brought on by OSA on the progression of myocardial infarction are likely offset by additional detrimental effects in the medium to long term. By causing the creation of neointima, it has been shown that HIF-1 may also contribute to the restenosis of coronary arteries after balloon angioplasty. Additionally, HIF-1 has been linked to atherogenesis, an inflammatory process that produces lipid-laden macrophages, intra-plaque angiogenesis, intra-plaque haemorrhage, and other processes. Treatment of OSA has been linked to improvements in endothelial dysfunction and decreases in circulating inflammatory markers [23], which suggests that it should slow the progression of atherosclerosis. A type of apnoea, central sleep apnoea (CSA), is uncommon in the general population but is frequently found in diseases like heart failure (HF), which are characterised by salt and water retention [26]. Only about 10% of people with clinically severe sleep apnoea symptoms are reportedly diagnosed. All healthcare professionals should be able to recognise and diagnose OSA syndrome and other sleep disorders, according to clinical studies and consensus panels. Primary healthcare providers also need to learn how to gather the necessary sleep histories for diagnosis [28]. Independent of body fat levels, sleep apnoea is linked to poor glucose tolerance and insulin resistance [32].

Mechanism of Action of CPAP Therapy

**Figure 3:**
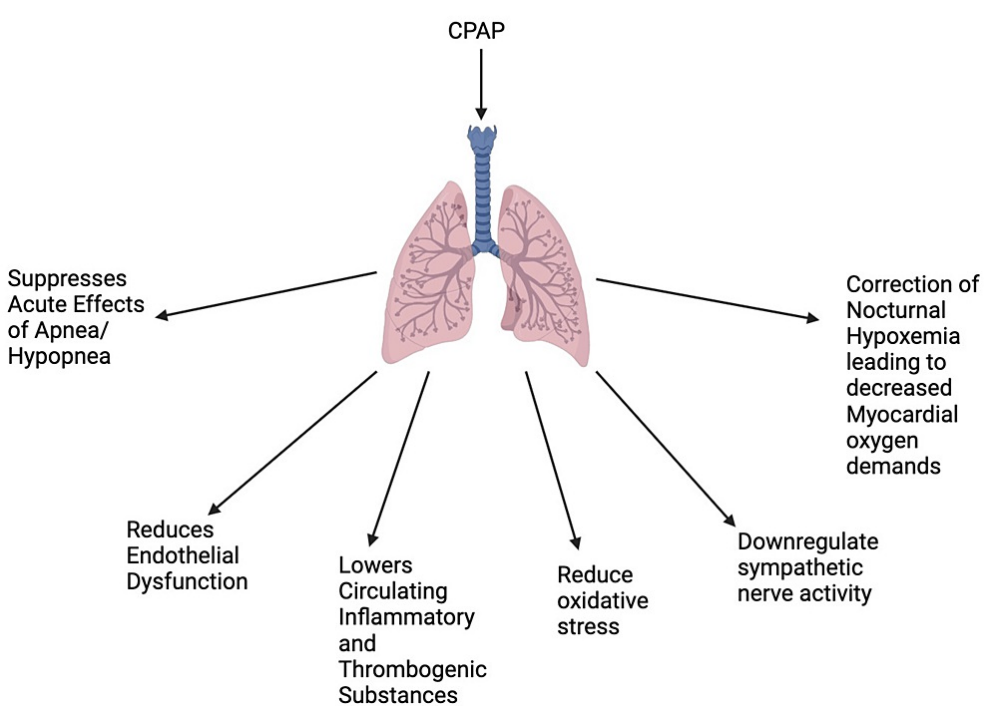
Illustrating the beneficial effects of continuous positive airway pressure. Created by Shivling Swami. CPAP: Continuous Positive Airway Pressure

CPAP therapy has been shown to improve symptoms and other surrogate outcomes, including blood pressure and glycemic management, by lowering hypoxemic burden and obstructive episodes. Theoretically, CPAP therapy is anticipated to lessen individuals with OSA's risk of cardiovascular events by obstructing the mechanisms mentioned earlier [1]. CPAP has been shown to alleviate endothelial dysfunction, lower circulating inflammatory and thrombogenic substances, reduce oxidative stress, and down-regulate sympathetic nerve activity. CPAP can slow the progression of cardiovascular diseases like atherosclerosis since it can rectify these dysfunctions [9]. Additionally, hypoxic episodes occur more frequently at night, making us wonder if CPAP's positive effects are attributable to suppressing the acute effects of apnoea-hypopnea [20]. A drop would reduce myocardial oxygen demands in sympathetic tone and blood pressure, and heart wall stress may be lessened by the correction of nocturnal hypoxemia and the attenuation of the negative intrathoracic pressure brought on by obstructive apnoea-hypopnea [23-25]. The current study demonstrates that treatment with CPAP reduced 24-hour mean arterial pressure in patients with previously undiagnosed moderate-to-severe OSA, even in a clinical setting where cardiovascular risk factors, including blood pressure, were well managed and despite the exclusion of patients with the most severe OSA who might be expected to benefit most from treatment. The treatment benefit was seen in individuals who were already taking antihypertensive medications, despite the fact that the average drop in blood pressure caused by CPAP was moderate compared to the effect of those drugs [27-29]. Overall patients with metabolic syndrome and OSA syndrome benefit from CPAP therapy's improvement of vascular dysfunction and reduction of oxidative stress [33,34]. The following beneficial effects have been illustrated below in Figure 3.

Analysis

We sought to determine the effect of CPAP on cardiovascular events in this updated systematic review of meta-analysis, RCT, systematic reviews, and meta-analysis, case-control studies and observational studies involving patients with OSA. The main findings of this analysis are (1) there was no significant difference between the CPAP and control groups in the occurrence of MACE at a mean follow-up of 42.6 months; (2) On subgroup analysis, CPAP use was associated with lower risk of MACE; (3) We discovered concerns about the included studies' potential for bias, primarily as a result of participant and staff blinding; (4) We discovered additional concerns about the applicability of the findings, primarily as a result of variations between included studies, including either non-sleepy or sleepy subjects, adherence to CPAP therapy, and the typical follow-up of the included RCTs; OSA can independently increase the risk of cardiovascular events, even after controlling for confounding factors, according to a systematic study conducted by Yingke Zhao et al. and observational study conducted by Yusel Peker et al. [14,17]. Due to its significant impact on cardiovascular problems, sleep health should be given top priority. According to Zhao et al. CPAP therapy may lower this risk if the equipment is used for at least four hours daily [18]. In the past, meta-analyses were undertaken to evaluate the role of CPAP in improving OSA patients' cardiovascular outcomes. Abuzaid et al. conducted a meta-analysis that comprised four randomised studies and found that utilising CPAP was not related to better cardiac outcomes than medical therapy alone, with the exception of patients who adhered to it (>four hours per night). A positive airway pressure device is not used by 20% to 40% of patients, and many others do not use it every night [12]. Similar findings were also shown in the meta-analysis by Khan et al. showing that CPAP positively affects cardiovascular events only in CPAP-adherent patients. The pathophysiology underlying cardiovascular complications such as heart attack and stroke in patients with OSA differs from that in patients with central sleep apnoea. Many patients with central sleep apnoea have significant left ventricle (LV) systolic dysfunction or stroke as an underlying condition, and they are at a heightened risk for additional cardiovascular events regardless of CPAP therapy. Therefore, combining OSA with central sleep apnoea may reduce the actual benefits of CPAP therapy. The benefit level from CPAP therapy would differ between OSA and central sleep apnoea. Our research revealed that CPAP was less associated with improved cardiovascular outcomes than medical therapy alone. In the most significant randomised trials on this subject and earlier meta-analyses, it was reported that CPAP had no impact in lowering cardiovascular events in OSA [8], however, Y. Chen et al. demonstrated a decreased risk of MACE by meta-analysis linked to CPAP, particularly in the subgroup with an Apnea Hypopnea Index (AHI) of less than 30 events/h. The findings were identical regarding individual outcomes, such as all-cause and cardiovascular death. Compared to control patients in the usual care group, CPAP is linked to a risk of MACE reduction of at least 37%. Their analysis, nevertheless, produced a negative result for myocardial infarction (MI), stroke, or repeat revascularization. However, the patients in this trial did not go to a sleep unit; instead, they were chosen from coronary units without being referred for any sleep-related complaints or breathing issues. It was challenging to achieve good CPAP therapy adherence in this population. Due to this population's deficient CPAP compliance (2.78 hours per night), the positive effects of long-term, properly administered CPAP (greater than or equal to four hours per night) could not be ruled out. It has been determined that patients with adequate adherence experience the effects more so than patients without acceptable adherence [9]. Similar results were found in a meta-analysis of RCTs by Jei YU et al. who discovered that the use of CPAP therapy had no discernible impact on cardiovascular deaths or events. However, it is expected to do so for several other sleep apnoea-related outcomes. According to the facts, it is fair to suggest CPAP therapy to relieve symptoms in OSA patients but not prevent vascular disease or death. It is conceivable to find protective effects of CPAP treatment for some patient subsets with an improved evidence base that can further study impacts in patient subgroups. These findings highlight the value of tried-and-true treatments for patients with sleep apnoea, which should be managed per established guidelines for patients at high cardiovascular risk, including blood pressure lowering, lipid-lowering, and anti-platelet therapy. Sleep apnoea is prevalent and is linked to multiple risk factors, including hypertension, obesity, insulin resistance, and diabetes. Although advantages in glycemic management and BMI have not been proven, CPAP has been shown to improve endothelial function, reduce insulin resistance, lower blood pressure, and increase insulin sensitivity and raise insulin sensitivity. A rationale for anticipating that adequately powered trials of CPAP would show beneficial effects on complex vascular outcomes was provided by the observed associations of sleep apnoea with vascular risk and the apparent beneficial effects of CPAP on intermediate biomarkers [7]. In contrast to this study, an observational study conducted by Roxena Pealva et al. has demonstrated that long-term CPAP therapy may contribute to body weight control in those with resistant hypertension and OSA. Despite the small study sample size, they revealed a significant reduction in mean BMI in the group of patients who adhered to long-term CPAP therapy. This is notable, the patients made no specific dietary or lifestyle adjustments. In the treatment group, they noticed a slight drop in BMI, whereas the control group's BMI increased [20]. Patients with CAD have an increased prevalence of OSA, and earlier observational cohort studies have shown a direct link between OSA and MACCEs in those patients. The first line of treatment for OSA is CPAP, which lessens daytime sleepiness in symptomatic patients. However, most adults with CAD with concurrent OSA do not report daytime sleepiness, and there is no agreement on CPAP therapy for these patients. According to a prior study, OSA patients who underwent percutaneous coronary intervention (PCI) and got CPAP had a lower cardiac death rate five years later than those who did not. In clinical cohorts, further observational studies have also revealed the positive cardiovascular effects of CPAP. However, the RCT in the Intention-To-Treat (ITT) populations [19,20] did not support these findings.This study demonstrated a neutral impact of routine CPAP prescription to people with acute coronary syndrome (ACS) and non-sleepy OSA on the rate of long-term cardiovascular events in the ITT group. After controlling for baseline co-morbidities and CPAP adherence, a significant protective impact of CPAP was initially identified. Furthermore, compared to ACS patients without OSA at baseline, patients with untreated/non-adherent OSA had a nearly two-fold increased risk for MACCEs [19]. The Sleep Apnoea Cardiovascular Endpoints (SAVE) trial is the largest trial to date conducted by Doug et al. including 2717 patients and the report revealed that routinely prescribing CPAP to CAD patients with non-sleepy OSA did not significantly reduce negative long-term outcomes in the ITT cohort. However, the risk reduction was seen once co-morbidities at baseline and CPAP adherence were taken into account. This secondary prevention trial found that adding CPAP therapy to conventional care did not lower the incidence of major cardiovascular events compared to standard care alone in people with cardiovascular disease and OSA. Treatment with CPAP was associated with improved health-related quality of life, mood, and job attendance in addition to a greater reduction in symptoms of daytime sleepiness. This investigation was not powered to provide definitive answers on the effect of CPAP on secondary cardiovascular endpoints, even though there was no evidence of significant advantage in relation to any cause-specific cardiovascular outcome. During a 44-month follow-up, the researchers found no statistically significant difference between the groups receiving CPAP and normal care in major adverse cardiac or cerebral events [6]. A notable design issue in this study is the fact that the diagnosis and treatment of sleep apnoea required to be more well-established in clinical practice before the investigation began. However, prior to trial recruitment, they invested a lot of time and effort in planning training sessions for investigators and study coordinators. According to the investigation, CPAP may help people who use it regularly prevent cardiovascular events. It is crucial to remember that this was based on a subgroup analysis and should be viewed as a finding that generates hypotheses. To ensure a high standard of research conduct, intensive site monitoring was also done throughout the trial. The average CPAP adherence reported in the study was only 3.3 hours per night, this was another potential drawback of this study. Another potential drawback is the apparent inadequacy of the CPAP dose for cardiovascular events. The trial population, which was 64% Asian, may have diluted the impact of the intervention. Another concern is the choice of an older "survivor" participant, with a mean age of 61 [6]. According to the available data, OSA may also be a predictive factor for heart failure, AF, MI and possibly renal impairment. The systematic review by Yingke Zhao et al. anticipated their investigation would reinforce the case for a significant role in OSA diagnosis and therapy in patients with cardiovascular disturbances and disorders. Patients with CAD have an increased prevalence of OSA, and earlier observational cohort studies have shown a direct link between OSA and MACCEs in those patients [14]. The first line of treatment for OSA is CPAP, which lessens daytime sleepiness in symptomatic patients. There is no consensus on CPAP therapy for people with concomitant CAD and OSA, and the majority of these patients do not report daytime sleepiness. An earlier study found that the five-year cardiac death rate was lower in OSA patients who underwent PCI and received CPAP than in those who did not. Additional observational studies have also demonstrated the beneficial cardiovascular benefits of CPAP in clinical cohorts. These conclusions, however, were not supported by the SAVE RCT trial in populations with an ITT design [19]. The multi-centre RICCADSA (Randomised Intervention with CPAP in CAD and OSA) trial, which examined the efficacy of CPAP with standard of care, involved 234 patients with newly revascularized CAD and OSA to address these issues [19]. The RICCADSA showed no meaningful difference between CPAP and the standard of care on long-term cardiovascular events, in line with other research. However, the authors found that at 57 months, individuals who used CPAP for under four hours each night experienced a significant reduction in cardiovascular events compared to those who did not use it or to the group of patients receiving standard care (2.3% versus 5.3%, respectively). Barbe et al. conducted a randomised trial (median four-year follow-up) evaluating CPAP (n = 357, mean age 52.0) versus no active ventilation (n = 366, mean age 51.8) in non-sleepy patients with newly diagnosed OSA who did not have prior cardiovascular disease, on incident hypertension or cardiovascular events. This study demonstrated a neutral impact of routine CPAP prescription to people with ACS and non-sleepy OSA on the rate of long-term cardiovascular events in the ITT group. After controlling for baseline co morbidities and CPAP adherence, a significant protective impact of CPAP was initially identified [2]. Furthermore, compared to ACS patients without OSA at baseline, patients with untreated/non adherent OSA had a nearly two-fold increased risk for MACCEs [19]. Soriano et al. conducted a post-hoc analysis of the studies by Barbe et al. [22]. A traditional polysomnographic or cardiorespiratory sleep study was used to diagnose OSA. The control group's mean AHI at baseline was 35, while the CPAP group was 42. The baseline Epworth Sleepiness Scale (ESS) for both groups was 6.5. The results of this study mean that compared to normal care, CPAP did not significantly reduce the occurrence of hypertension or cardiovascular events. In contrast to standard care, this showed that patients with moderate to severe OSA (AHI > 20/h) without daytime sleepiness did not experience a significant reduction in the incidence of cardiovascular events over a four-year period of follow-up [22]. However, Barbe et al. found that CPAP had a marginally beneficial effect on cardiovascular events only in patients who used it for four hours or longer each night [2]. The Guo J et al. meta-analysis found no significant treatment benefit of CPAP on mortality, stroke, and cardiovascular events compared with the control groups, however, mortality and incidence of cardiovascular events tended to decrease following CPAP therapy compared to placebo treatment. Despite having no discernible effects on BMI, CPAP therapy significantly decreased ESS, a symptom of less tiredness. The CPAP group also saw significantly lower systolic blood pressure (SBP) and diastolic blood pressure (DBP). Overall, CPAP therapy was linked to a lower risk of cardiovascular events, and given the therapeutic benefit, it is a safe and effective treatment for individuals with OSA. Additionally, considerably lower in the CPAP group were ESS and BP [13]. CPAP compliance is a significant factor in many studies where inadequately treated OSA. The timing of CPAP may also be significant, which is not frequently reported in trials, since two of the evaluated trials demonstrated improvement in cardiovascular endpoints with adjusted analyses in the more asymptomatic patients (CPAP use four-hour nightly). Compared to later or early morning when rapid eye movement (REM) sleep predominates, CPAP use in the evening may be less beneficial. Compared to apnoea or hypopnea events during non-REM sleep, REM sleep episodes are longer (including more oxygen desaturation) and more strongly related to hypertension. The outcomes of OSA trials frequently need help with their generalisability. Most studies omit older and sleepier groups in favour of younger male volunteers. Additionally, non-sleep individuals respond less favourably to CPAP. One explanation is that patients who are not asleep may be more inclined to disregard CPAP instructions [15]. According to Miguel Angel Martinez et al., the systematic review conducted by them showed that there is clinical proof that OSA increases the risk of developing systemic hypertension and is poorly managed. However, due to the complex nature of systemic hypertension, there has been much variation in the results regarding the impact of CPAP therapy on blood pressure. As a result, there is a growing interest in researching patient subgroups who might profit from CPAP therapy. Despite other confounding factors like obesity, OSA is very common in individuals with resistant hypertension, indicating that this subgroup of hypertensive patients may benefit from CPAP therapy. According to international standards, a reduction in blood pressure, which is a risk factor for both coronary heart disease and stroke in the order of two to three millimetres of mercury (mm Hg) of SBP, could dramatically cut ultimate cardiovascular mortality (between 6% to 8% for stroke and 4% to 5% for coronary heart disease). There have only been a few studies evaluating the efficacy of CPAP therapy in patients with resistant hypertension and OSA. According to the research that is now available, BP decreases that are clinically significant have been found, especially at night and in patients who adhere to their CPAP therapy successfully. Particularly at night and in those patients, there is a clinically and statistically substantial decline in 24-hour mean and DBP. These studies' substantial methodological problems, such as the small cohort sizes and lack of randomisation, prompted their authors to emphasise the need for more in-depth research [16]. However, a more thorough investigation or a longer length of follow-up might have been able to establish a significant connection between the course of therapy and the outcome. A blog article assessing this study claimed that those who used CPAP for more than four hours every night benefited. Compared to past observational studies, the degree of the effect was lower in these patients. Another possibility is that those who are not sleeping have lower CPAP effectiveness. In short-term studies focusing on awake subjects, clinical symptoms or blood pressure were not improved by CPAP, and only those patients who used CPAP for at least 5.6 hours each night noticed a moderately positive improvement. Despite being more obese and having a higher frequency of hypertension, the subgroup of patients in this research who adhered to their CPAP therapy for four hours or longer each night displayed a protective association with the occurrence of hypertension or cardiovascular events. However, post hoc study findings should be taken with a grain of salt and used to form hypotheses. In these studies, the patients were not randomised into the subgroups. The healthy-user effect, for example, may have caused results to be skewed because CPAP users who follow their routine are more likely to follow other treatment regimens, dietary recommendations, or other health maintenance regimens. Similar findings were found in an RCT by Zhiwei Huang et al. which demonstrated that long-term CPAP therapy significantly decreased daytime SBP and improved hypertension control but did not further lower daytime DBP in hypertensive patients with coronary heart disease (CHD) and OSA receiving conventional antihypertensive therapy. Studies showed a decreasing tendency in the CPAP group. In the CPAP group, daytime somnolence caused by OSA was dramatically reduced compared to controls. Obesity is an independent risk factor unaffected by CPAP and may help decrease BP control [4]. Additionally, the trial only lasted four weeks. Similar findings were found in observational research by Ryutaro Shirahama et al. which included 918 OSA patients. Compared to patients with poor CPAP adherence, we discovered a substantial drop in DBP among those with good CPAP adherence throughout the 24-month follow-up period (= 0.13, p = 0.03). Weight loss and CPAP use were not significantly associated (= 0.02, p = 0.59). Lowered DBP without appreciable weight reduction was linked to long-term, good CPAP therapy adherence [21]. The most extraordinary sample size was used in a multicenter, RCT by Barbé et al. which demonstrated a modest drop in blood pressure. However, this study did not discover an appreciable decrease in the incidence of hypertension with CPAP therapy in OSA patients without hypertension [2]. The fact that the Individuals were all non sleepy hypertensive patients may have contributed to the disparate results. Additionally, only one patient in the CPAP group experienced an Severe Cardiovascular and Cerebrovascular Events (SCCE) compared to five individuals in the control group who had SCCEs. One of the five individuals passed away from acute myocardial infarction (AMI). Although no difference was found, the statistics are encouraging. A statistical difference might have been seen with a longer follow-up and larger sample size. The OSA and CHD populations will be greatly impacted if the rumour is true [4]. Soriano et al. evaluated the cardiovascular events in 163 OSA patients based on CPAP use in a post hoc analysis of the trial. After 58 months of follow-up, they found that patients who used CPAP for under four hours every night had a threefold lower incidence of cerebrovascular events. According to some authors, the number of hours spent using a CPAP machine determines how well it lowers blood pressure [22]. This study demonstrates a substantial correlation between the amount of CPAP sessions utilised (especially in patients utilising it for at least four hours each night) and the reduction in blood pressure levels, which lends weight to this conclusion. In line with other significant investigations of individuals with OSA, more than 70% of participants in the current study adhered to their CPAP therapy for four or more hours every night [22,16]. The results of earlier meta-analyses' subgroup analyses likewise pointed to a comparable advantage for CPAP use in preventing major cardiac events in OSA patients. Similar findings were reached in a meta-analysis by Felipe da Silva Paulitsch et al. who found that a substantial problem and the main reason for CPAP's ineffectiveness on survival and cardiovascular outcomes lack non-OSA patients is poor treatment adherence [11]. Propensity-score matched analysis of the SAVE trial found that relative to usual care, four hours of CPAP use per night was associated with a significantly lower risk of stroke and the non-pre specified composite endpoint of cerebral events, but not primary composite cardiovascular events. The secondary on-treatment analysis of another trial revealed a significant effect of four hours of CPAP use per night compared to fewer than four hours per night or no CPAP use in lowering the risk of cardiovascular events in individuals with non-sleepy OSA and cardiovascular disease (CVD). However, many comparisons and persisting self-selection bias might have affected such "positive" results. The sensitivity studies in the current meta-analysis and the subgroup analyses in the previous meta-analysis failed to consistently show the effects of prolonged CPAP use (four hours per night) on cardiovascular outcomes in people with sleep apnoea. It is unclear how enhanced CPAP therapy compliance may affect cardiovascular risk reduction and what level of PAP use per night is necessary [11]. The results of case-control research conducted by Francisco Garcia-Rio et al. show that OSA and MI have an independent relationship. Furthermore, it has been demonstrated that OSA patients who tolerated CAD patients without OSA who used CPAP had a lower incidence of recurrent MI and revascularization. This study also found that patients with CAD and OSA who were using CPAP experienced fewer recurrent episodes and required fewer revascularization procedures than those who were not using CPAP. According to some earlier research, CPAP may help these individuals experience fewer adverse severe cardiac or cerebrovascular events [23]. The lower risk for recurrent MI and new revascularization procedures found in the patients who received CPAP suggests that this treatment may affect myocardial microcirculation. However, the risk of recurrent MI or the need for revascularization had not been considered as specific endpoints but was instead included in composite endpoints. Considering the widespread use of antiplatelet therapy, drug-eluting stents, and lipid- and blood-pressure-lowering medications in the modern day, treating OSA with CPAP may not provide further benefits for CAD patients. When examining patients with CAD in clinical practice, we should still pay greater attention to OSA because it is so common and is linked to a higher risk of cardiovascular disease. Further research is required to determine whether improved CPAP compliance or innovative treatment approaches can improve cardiovascular outcomes [5]. Despite the lack of evidence, future research examining these pertinent outcomes is required [10]. Although there is no proof that CPAP therapy improves CV outcomes such as stroke, hypertension and MI, concerns about the risk of bias, CPAP adherence, and the population included in each RCT may have diminished the strength of the findings to support the benefit in all patients. 

Strengths and Weaknesses

One of the key strengths of our analysis is the inclusion of a subgroup analysis based on CPAP adherence. This additional analysis allows us to examine the potential benefits of CPAP therapy when patients consistently adhere to treatment. We found that among the CPAP-adherent subgroup, there was a significant reduction in MACE, suggesting that adherence to CPAP treatment plays a crucial role in achieving positive outcomes. These findings highlight the importance of patient education and support to promote long-term adherence to CPAP therapy. The lack of an overall benefit for CPAP therapy in our analysis should be interpreted while considering certain limitations in the included randomised trials and other research studies. First, the majority of studies have mostly included non-severe OSA. Prior observational studies suggested that patients with severe OSA carry the highest risk of cardiovascular events; hence, the magnitude of the benefit of CPAP could be underestimated by eliminating those patients. Second, the magnitude of benefit from CPAP might differ in primary prevention versus secondary prevention in patients with OSA. Most randomised trials included patients with established cardiovascular events, and high cardiac risk factors, in whom OSA might not play a substantial risk factor compared to lower-risk populations. Therefore, the role of CPAP in the primary prevention of cardiovascular events cannot be determined from the current randomised data. Also, the included studies exhibited heterogeneity regarding patient characteristics, such as age, sex, and comorbidities. Therefore, caution should be exercised when extrapolating the results to broader populations. Future studies should include more diverse patient populations to enhance the external validity of the findings. Additionally, the issue of CPAP adherence poses a challenge in real-world clinical settings. While our subgroup analysis suggests that adherence is associated with improved outcomes, it is essential to recognise that achieving and maintaining adherence can be difficult for some patients. Further research should focus on identifying effective strategies to enhance CPAP adherence, such as personalised patient education, regular follow-up, and addressing barriers to adherence. Our review also identified a potential risk of bias in the included studies, particularly related to the blinding of participants and personnel. Blinding is challenging in CPAP studies due to the nature of the intervention, and the lack of blinding may introduce performance and detection bias. It is crucial for future research to implement rigorous blinding methods and consider alternative study designs, such as randomised controlled trials, to minimise bias and strengthen the evidence base. Finally, many of the included studies suffered poor adherence to CPAP therapy among the included patients. Our subgroup analysis is hypothesis-generating and suggests that adherence to CPAP may play an important role in determining the impact of CPAP on cardiovascular outcomes. We hypothesize that achieving a clinically meaningful reduction in cardiovascular events mandates the application of CPAP four hours or more daily to reverse airway obstruction, hypoxemia and ensuing detrimental mechanisms. In conclusion, our systematic review provides important insights into the impact of CPAP therapy on cardiovascular events and mortality. While the findings suggest a potential benefit, caution is warranted in generalising the results due to the heterogeneity of the included studies and the challenges associated with CPAP adherence. By addressing these limitations and implementing our proposed recommendations, future research can contribute to a more comprehensive understanding of the role of CPAP therapy in improving patient outcomes.

Limitations

Statistical heterogeneity in outcome measures of MACE exists due to different study designs, small sample sizes, and variations in study quality and adjustment for confounders. Study populations vary from general CAD patients to specific subsets with MI and revascularization. Future studies should focus on more homogeneous patient groups. Differences in definitions and adherence to CPAP treatment impact treatment effects compared to control groups. Standardized definitions are needed. Risk estimates of individual cardiovascular events could be more balanced due to the small number of studies and variations in event definitions. Summary data from moderate-to-large-sized RCTs show limited events and may not capture the moderate benefits of positive airway pressure (PAP) therapy. More data are needed to detect these effects. Adverse effects of PAP therapy may be revealed with additional data, as some associations suggest harm rather than benefit. Recommendations for future studies: In light of the limitations identified in our review, we propose several recommendations for clinical practice and future research. Firstly, healthcare professionals should prioritise patient education and support to improve CPAP adherence. Overall, better-designed randomised trials are warranted to appreciate the true role of CPAP therapy on cardiovascular events among patients with OSA. Future trials should pursue alternate designs to maximise CPAP adherence rates among participants and, importantly, enrol sleepy patients with OSA who would hypothetically benefit more from CPAP therapy. This may involve providing clear instructions on CPAP use, addressing patient concerns and misconceptions, and offering ongoing support and monitoring. Furthermore, given the potential impact of patient characteristics on treatment outcomes, clinicians should consider individualised approaches when prescribing CPAP therapy. Future research should focus on conducting well-designed RCTs encompassing diverse patient populations and address the limitations of the current evidence. Additionally, investigations into the long-term effects of CPAP therapy, the optimal duration of treatment, and the comparative effectiveness of different CPAP devices and modes of delivery would provide valuable insights into refining treatment strategies. Standardisation is crucial to reduce heterogeneity in study design, CPAP application, sleep evaluation, and event definitions. This will enhance comparability and reliability across studies. focusing on more homogeneous patient groups will enable a more accurate assessment of CPAP treatment effects. Large sample sizes should be considered to adequately power the analysis and detect statistically significant effects on cardiovascular endpoints. Additionally, longer follow-up periods are necessary to accurately assess the clinical benefits of CPAP therapy. Comparative placebo-controlled trials can help reduce bias and provide stronger evidence for treatment effects. Thorough documentation of potential confounders, co morbidities, and clinical variables is essential in cohort studies to account for their influence on prognosis. Homogenous patient populations, such as those with CAD or MI, should be the focus of research to accurately evaluate the treatment effects of CPAP therapy. Increasing sample sizes will provide adequate statistical power for detecting meaningful differences in cardiovascular events. Extending the duration of follow-up will capture long-term outcomes and comprehensively evaluate the clinical benefits of CPAP therapy. Incorporating placebo-controlled arms in trials is important to assess the true efficacy of CPAP therapy and reduce bias. Lastly, establishing standardized protocols, encouraging collaboration and data sharing, and performing sensitivity analyses will enhance the reliability and generalizability of study findings. By implementing these recommendations, future research on CPAP therapy can be improved and provide more robust evidence for its effectiveness. By implementing these recommendations, future studies can address the limitations identified and provide more robust evidence regarding the effectiveness and safety of CPAP therapy in cardiovascular outcomes.

## Conclusions

Even though our present study indicates that patients with a diagnosis of cardiovascular disease and OSA do not benefit from CPAP therapy when it comes to preventing clinically relevant outcomes like mortality or improving cardiovascular disease, it is essential to remember that the absence of any effect is not equivalent to the absence of an effect. In our evaluation, we highlight several issues with most research, how to read these studies and this meta-analysis. (1) Even though CPAP therapy did not reduce the risk of cardiovascular events, these results should be interpreted cautiously, and further study is required before RCTs involving patients with OSA are designed differently. (2) Long-term follow-up shows a high correlation between CPAP adherence and better outcomes. Additional research should concentrate on incorporating OSA patients who use CPAP for more hours per night as few studies mentioned in the analysis section described that the normal patients were using CPAP only for 3.3 to 3.5 hours per night which might not be enough to unveil the true positive effects of CPAP, so studies where patients have used CPAP for four hours or more than four hours per night should be included. (3) Different OSA phenotypes, including subgroups like sleepy vs. non-sleepy patients, hypoxemic characteristics in patients with moderate to severe OSA, and descriptions of poorer populations using metrics other than the AHI, should all be included in novel RCTs. (4) The majority of studies involved patients from hospital and sleep clinic populations, and future research should include population-based studies to examine the effectiveness in the primary prevention of cardiovascular illnesses.
